# Effect of Four Functional Feed Additives on Growth, Serum Biochemistry, Antioxidant Capacity, Gene Expressions, Histomorphology, Digestive Enzyme Activities and Disease Resistance in Juvenile Olive Flounder, *Paralichthys olivaceus*

**DOI:** 10.3390/antiox12081494

**Published:** 2023-07-26

**Authors:** Wonsuk Choi, Mohammad Moniruzzaman, Ali Hamidoghli, Jinho Bae, Seunghyung Lee, Seunghan Lee, Taesun Min, Sungchul C. Bai

**Affiliations:** 1 Feeds & Foods Nutrition Research Center, Pukyong National University, Busan 48547, Republic of Korea; hm622@naver.com (W.C.); ali.hamidoghli@yahoo.com (A.H.); 2 Department of Animal Biotechnology, Jeju International Animal Research Center, Sustainable Agriculture Research Institute (SARI), Jeju National University, Jeju 63243, Republic of Korea; monir1983@jejunu.ac.kr; 3 Aquafeed Research Center, National Institute of Fisheries Science, Pohang 53717, Republic of Korea; bjh2921@naver.com (J.B.); shlee5863@naver.com (S.L.); 4 Major of Aquaculture and Applied Life Sciences, Division of Fisheries Life Sciences, Pukyong National University, Busan 48513, Republic of Korea; shlee@pknu.ac.kr; 5 Department of Animal Biotechnology, Bio-Resources Computing Research Center, Sustainable Agriculture Research Institute (SARI), Jeju National University, Jeju 63243, Republic of Korea; 6 FAO World Fisheries University Pilot Program, Busan 48547, Republic of Korea

**Keywords:** yeast-extracted nucleotide, peptide, taurine, mineral water, growth performance, antioxidant defense, gene expressions, histology, disease resistance, olive flounder

## Abstract

An 8-week feeding trial was executed to evaluate the efficacy of four functional feed additives in replacing antibiotics in juvenile olive flounder, *Paralichthys olivaceus*, fed with a low-fish-meal diet. A basal diet without feed additives was used as a control (CON); other diets were formulated by supplementing 0.50% taurine (TW), 0.30% peptide (PT), 0.23% mineral water (MW), 0.35% yeast-extracted nucleotides (GRO), 0.35% GRO + 0.50% taurine (GROTW), 0.35% GRO + 0.30% peptide (GROPT) and 0.35% GRO + 0.23% mineral water (GROMW) into the basal diet; in addition, one diet was supplemented with oxytetracycline (OTC) at 0.5% as a positive control. Triplicate groups of 25 fish with an average weight of 5.15 ± 0.06 g (mean ± SD) were fed one of the nine experimental diets. At the end of the feeding trial, the weight gain, specific growth rate and protein efficiency ratio of fish fed the GRO, GROMW, GROPT and GROTW diets were significantly higher than those of fish fed the CON diet (*p* < 0.05). The feed efficiency of fish fed the GRO, GROMW, GROPT and GROTW diets was significantly higher than that of fish fed the TW and OTC diets. However, the survival, hepatosomatic index, viscerosomatic index and condition factor of fish, as well as their whole-body proximate composition, were not significantly affected by the experimental diets (*p* > 0.05). The serum glutamic pyruvic transaminase of fish fed the GROPT diet was significantly lower than that of fish fed the CON diet. However, glutamic oxaloacetic transaminase, glucose and total protein were not significantly affected by the experimental diets (*p* > 0.05). The serum superoxide dismutase activity of fish fed the PT, TW, GRO, GROMW, GROPT and GROTW diets was significantly higher than that of fish fed the CON diet. The lysozyme activity of fish fed the PT, GRO, GROMW, GROPT and GROTW diets was significantly higher than that of fish fed the CON and OTC diets. The myeloperoxidase activity of fish fed the TW, GRO, GROMW, GROPT and GROTW diets was significantly higher than that of fish fed the CON, PT and MW diets (*p* < 0.05). The flounder growth hormone gene expression of fish fed the TW, GRO, GROMW, GROPT, GROTW and OTC diets was significantly higher than that of fish fed the CON, PT and MW diets (*p* < 0.05). The interleukin 1β and interleukin 10 gene expressions of fish fed the GRO, GROMW, GROPT and GROTW diets were significantly higher than those of fish fed the CON, PT, TW and MW diets (*p* < 0.05). Intestinal histology showed a significantly higher villi length for fish fed the GRO, GROMW, GROPT and GROTW diets compared to that of fish fed the CON diet (*p* < 0.05). Digestive enzyme activities such as trypsin activity were significantly higher in fish fed the GROMW, GROPT and GROTW diets than those in the rest of the diet groups (*p* < 0.05). Amylase activity in fish fed the MW, GRO, GROMW, GROPT, GROTW and OTC diets was significantly higher than that of fish fed the PT, TW and CON diets (*p* < 0.05). On the other hand, the lipase activity of fish fed the TW, GRO, GROMW, GROPT and GROTW diets was significantly higher than that of fish fed the CON, PT, MW and OTC diets (*p* < 0.05). The cumulative survival rate of fish fed the PT, GROTW, GROPT and GROMW diets was significantly higher than that of fish fed the CON, TW and MW diets after thirteen days of the challenge testing. Overall, the results demonstrate that the GRO, GROMW, GROPT and GROTW diets could be beneficial feed additives to replace antibiotics in juvenile olive flounder fed low-fish-meal diets.

## 1. Introduction

Aquaculture has developed substantially in recent decades, with production rising from 31 million metric tons (mt) in the year 2000 to 81 mt in 2018 [1]. This steady and rapid growth has introduced aquaculture to the world as the fastest growing animal-protein-producing sector, compared to beef, poultry and pork production [2]. Thus, it is expected that aquaculture will play an important role in global food safety, considering the anticipated population increase in the next few decades [3]. However, the sustainable development of aquaculture may be limited by new social, economic and environmental challenges. In developing countries with small-scale aquaculture, issues such as water quality, water resources, landscape changes, production costs, labor welfare, and marketing are major issues [4]. In developed countries with modernized aquaculture, the degradation of habitats, rural futures and economic restructuring, the impacts on wild stocks and species, environmental pollution and product quality have generated controversy [5].

One of the issues that has received increasing economic, environmental and social awareness in the sustainable development of aquaculture is the excess use of marine ingredients in fish feed [2]. Protein is the dominant and most expensive component in aquafeeds and is required for the maintenance and growth of animals. Generally, fish and shrimp require 25–50% protein in their diets, which is sourced from plant and animal ingredients such as soybean meal and fish meal [6,7]. Fish meal has been traditionally used as the main protein source in aquaculture feed due to its rich amino acid, nucleotide, essential fatty acid, phospholipid, mineral, vitamin, and fat content, and also because of its high palatability and digestibility [8,9]. The upsurge in the usage of fish meal in aquafeeds alongside the fast expansion of aquaculture production has increased the demand for this ingredient. However, world fish meal production is limited due to the overexploitation of natural resources. High demand and low resources have resulted in increased prices for this valuable ingredient [10]. Static supply, increasing price, and ethical considerations have forced the industry to reduce the inclusion level of fish meal in aquafeeds [11,12]. As an alternative to fish meal, several plant and animal protein sources such as soybean meal, corn gluten meal, meat and bone meal, and poultry by-products have been tested in the diets of fish and shrimp species [7,13,14,15,16]. However, high inclusion of these ingredients in aquafeeds is limited due to an imbalance in essential amino acids, the presence of antinutritional factors, inconsistent quality and low palatability and digestibility [2,9,17,18]. Recent studies have shown that some of the problems associated with alternative plant and animal ingredients can be partly addressed by the incorporation of functional feed additives in the diet [19].

Functional feed additives are defined as nutritive/non-nutritive compounds that are included in feed formulations to serve a specific function, such as improving the physical or chemical properties of the diet or the performance and quality of fish and their resulting products [18,19,20]. There are a variety of feed additives, quite diverse in their chemical nature, being used in feed formulations [19]. As an example, Bae et al. [21] recently reported that functional feed additives such as yeast extract nucleotides, gamma-aminobutyric acid and song-gang stone could enhance growth, feed utilization, antioxidant capacity, intestinal morphology and disease resistance in juvenile olive flounder fed a low-fish-meal diet. Furthermore, Wang et al. [22] found that supplementation with poultry by-product meal and selenium yeast increased fish performance compared to meal replacement with soybean meal in golden pompano. In the present experiment, peptide, taurine, mineral water and nucleotides were used and compared as potential feed additives in low-fish-meal diets. Peptides are short-chain amino acids connected to each other via peptide bonds and are distinguished from regular proteins by their shorter length. Antimicrobial peptides are important components that play a significant role in the innate immune systems of most species [23,24]. Taurine (2-aminoethanesulfonic acid) is an amino acid that exists naturally in mammals, birds, fish and aquatic invertebrates [25]. As a feed additive, taurine can affect bile salt formation, membrane stability, immunomodulation, anti-oxidation and mitochondrial function, and calcium signaling [26]. Kim et al. [25] and Jo et al. [27] reported that dietary supplementation with taurine can increase the growth and digestive physiology of juvenile and growing olive flounder fed low-fish-meal diets. Furthermore, dietary taurine was found to improve growth and feed efficiencies in rainbow trout fed a soy protein concentrate diet instead of fish meal [28]. Minerals have essential roles in various life processes in animals, including fish [29]. Out of about 90 elements, 22 elements are known to be essential for living beings [30]. Fish have a unique physiological mechanism that enables them to absorb and retain minerals from their diets and water [29]. Dietary supplementation with minerals has been shown to enhance growth performance, non-specific immunity and disease resistance in fish [31]. Nucleotides are intracellular compounds with a low molecular weight and play an important role in almost all biochemical processes in vertebrates and invertebrates [32]. They are non-essential nutrients in fish feed because they can be synthesized in the animal body. However, during challenging conditions such as stress, injury, nutrient deficiency, and early life stages, they seem necessary in the diet [33]. Therefore, nucleotides are regarded as conditionally essential nutrients [34]. Bae et al. [35] reported that a dietary purine-based nucleotide, inosine monophosphate (IMP), together with shrimp soluble extract (SSE) demonstrated a positive impact on growth, immunity and disease resistance in juvenile Nile tilapia. Furthermore, IMP and SSE were found to be easily obtainable from waste valorization processes, which would be economically feasible and involve fast production at lower costs. In a recent study, Choi et al. [36] found that a dietary yeast-extracted nucleotide with probiotics could replace antibiotics in terms of improving growth, immunity, gut health and disease resistance in juvenile olive flounder. These single-cell protein meals have a remarkably high protein content, with a well-balanced essential amino acid profile that does not compromise growth performance, and improve gut health and reduce enteritis associated with high-soybean feeds without being detrimental to the growth, antioxidation or immunity of fish. All the mentioned feed additives have shown great potential to enhance the growth and immune responses of fish [21,31,35,36].

Olive flounder *Paralichthys olivaceus* is a marine carnivorous fish species and the most cultured fish species in the Republic of Korea, and has high economic value throughout East Asia [1]. However, limited success has been achieved in the formulation of sustainable feed as olive flounder commercial feed contains more than 60% fish meal [37]. Therefore, the aim of the present study was to evaluate four types of functional feed additive (alone or in combination) in a low-fish-meal diet for olive flounder based on growth, serological indices, antioxidant enzyme activities, growth- and immune-related gene expressions, digestive enzyme activities and disease resistance.

## 2. Materials and Methods

### 2.1. Ethics Statement

This experiment was conducted under the guidelines of Institutional Animal Care and Use Committee Regulations, No. 554, issued by the Pukyong National University, Busan, Republic of Korea. Every effort was taken to minimize the number of fish sacrificed.

### 2.2. Experimental Diets

The basal diet formulation is shown in Table 1. Sardine (68.39% CP) and anchovy fish meal (68.75% CP) along with soybean meal (47.04% CP) were used as the main protein sources, while fish oil was used as the main lipid source. The feed additives used in this experiment were a control (CON) without additive supplementation, taurine (TW, 0.5%), peptide (PT, 0.3%), mineral water (MW 0.217%), Gro-pro^®^ (GRO 0.35%), Gro-pro + taurine (GRO + TW 0.35% + 0.5%), Gro-pro + peptide (GRO + PT 0.35% + 0.3%), Gro-pro + mineral water (GRO + MW 0.35% + 0.217%) and oxytetracycline (OTC 0.5%). The dietary inclusion levels of four feed additives were based on previous studies, such as Choi et al. [36] for Gro-pro^®^ (GroPro Aqua, Angel Yeast Co. Ltd., Yichang, China), which is a yeast-derived protein and nucleic acid-based commercial feed additive used as a growth and palatability enhancer in aquaculture, and Kim et al. [25] and Jo et al. [27] for taurine. Additionally, according to the manufacturer’s recommendation, peptide, which is rich in peptide, nucleotide, glutamic acid, amino acids and inositol (NuPro, Alltech Inc., Seoul, Republic of Korea), and laboratory-made mineral water (consisting of 95% water and 5% minerals such as Ca, P, Na, K, etc.), were supplemented into the diets. The procedures for feed manufacturing and preparation were followed as previously described by [36]. According to the feed formulation table, all fine-powdered ingredients were mixed thoroughly using an electric mixer (HYVM-1214, Hanyoung Food Machinery, Hanam, Republic of Korea). Then, a stiff dough was formed by adding fish oil and the desired amount of water. The dough was passed through a pellet machine (SFD-GT, Shinsung, Seoul, Republic of Korea) using a 0.2 cm die. The prepared diets were air dried in a drying room for 48 h, and then, broken into smaller sizes and stored at −20 °C. According to the proximate composition analysis, shown in Table 2, all diets were iso-nitrogenous and iso-lipidic.

### 2.3. Experimental Fish and Feeding Trial

Juvenile olive flounder were obtained from a private farm (Jungang Fisheries, Chungcheongnam-do, Taean-gun, Republic of Korea). Prior to the start of the feeding trial, the health status of the fish was checked via visual observation on arrival and they were starved for 24 h. All the fish were fed the commercial diet for two weeks to enable them to become acclimatized to the laboratory conditions. A total of 675 fish averaging 5.15 ± 0.06 g (mean ± SD) were weighed and randomly distributed (25 fish/40 L tank) into twenty-seven indoor fiberglass tanks with a 40 L volume receiving a constant flow (1.2 L/min) of filtered seawater. Each tank was then randomly assigned to one of three replicates of nine dietary treatments. During the 8 weeks of the experiment, supplemental aeration was provided in each tank to maintain enough dissolved oxygen near saturation, and the water was also heated using electric heaters in a concrete reservoir. The water temperature and pH were maintained at 19.0 ± 1.0 °C and 7.5 ± 0.5, respectively, throughout the experiment. Fish were fed twice daily (09:00 and 19:00 h) for 8 weeks at a rate of 2~4% body weight per day. Dead fish were removed immediately and weighed, and the amounts of feed in the respective tanks were adjusted accordingly. Uneaten feed was siphoned out after 1 h of feeding, and the insides of the tanks were scrubbed once per week to minimize algal and fungal growth.

### 2.4. Sample Collection and Analysis

At the end of the feeding trial, fish were starved for 24 h, and the total number and weight of fish in each tank were determined to calculate the final weight (FW), weight gain (WG), specific growth rate (SGR), feed efficiency (FE), protein efficiency ratio (PER) and survival rate (SUR). Four fish per tank were randomly selected and individually weighed, and then, the same fish were anesthetized with ethylene glycol phenyl ether (200 mg/L for 5–10 min) and dissected to obtain the liver and gastro-intestinal organs for the determination of the hepatosomatic index (HSI) and visceral somatic index (VSI). Thereafter, the intestines from the same fish were used for further histological observation and enzyme activity. Three additional fish per tank were randomly captured and anesthetized with ethylene glycol phenyl ether (200 mg/L for 5–10 min), and blood was collected via injection with caudal puncture. Finally, the serum was separated via centrifugation at 5000× *g* for 10 min and stored at −70 °C for the analysis of non-specific immune response parameters, including lysozyme activity, superoxide dismutase (SOD) and myeloperoxidase (MPO) activities, and biochemical parameters, including glutamic pyruvic transaminase (GPT), glutamic oxaloacetic transaminase (GOT), glucose and total protein (TP). The serum levels of GPT, GOT, glucose and total protein were determined using a chemical analyzer (Fuji DRI-CHEM 3500i, Fuji Photo Film Ltd., Tokyo, Japan) following the manufacture’s instructions.

Three additional fish from each tank were randomly captured and used for analyses of whole-body proximate composition. Proximate composition analyses of the experimental diets and fish bodies were performed using the standard methods of the AOAC [38]. Samples of the diets and fish were dried at 105 °C to a constant weight to determine their moisture content. Ash content was determined via incineration at 550 °C. Protein was determined using the Kjeldahl method (N × 6.25) after acid digestion, and crude lipid was measured via Soxhlet extraction using the Soxhlet system 1046 (Tacator AB, Hoganas, Sweden) after freeze-drying the samples for 20 h.

### 2.5. Non-Specific Immune Response Analysis

The lysozyme activity was analyzed as follows: in brief, a test serum (0.1 mL) was added to 2 mL of a suspension of *Micrococcus lysodeikticus* (0.2 mg/mL) in a 0.05 M sodium phosphate buffer (pH 6.2). The reactions were carried out at 20 °C and absorbance at 530 nm was measured between 0.5 min and 4.5 min using a spectrophotometer. A lysozyme activity unit was defined as the amount of enzyme producing a decrease in absorbance of 0.001/min. Superoxide dismutase (SOD) activity was measured using the superoxide radical-dependent reaction inhibition rate of enzymes with WST-1 (water-soluble tetrazolium dye) substrate and xanthine oxidase using the SOD Assay Kit (Sigma-Aldrich, 19160, St. Louis, MO, USA) according to the manufacturer’s instructions. Each endpoint assay was monitored with absorbance at 450 nm (the absorbance wavelength for the colored product of the WST-1 reaction with superoxide) after 20 min of reaction time at 37 °C. The percentage inhibition was normalized by the mg protein and expressed as SOD unit/mg. Myeloperoxidase activity was measured according to the method described by Quade and Roth [39]. Briefly, 20 µL of serum was diluted with HBSS (Hanks Balanced Salt Solution) without Ca^2+^ or Mg^2+^ (Sigma-Aldrich, St. Louis, MO, USA) in 96-well plates. Then, 35 µL of 3, 3′, 5, 5′ tetramethylbenzidine hydrochloride (TMB, 20 mM) (Sigma-Aldrich, St. Louis, MO, USA) and H_2_O_2_ (5 mM) was added. The color change reaction was stopped after 2 min by adding 35 µL of 4 M sulfuric acid. Finally, the optical density was read at 450 nm using a microplate reader.

### 2.6. Bacterial Infection Test

After sampling, seven fish from each tank were redistributed into 27 tanks in a non-recirculating system without water renewal to perform the 9-challenge test based on the dietary treatments. The pathogenic bacteria *Edwardsiella tarda* FSW910410 were obtained from the Department of Biotechnology, Pukyong National University, Busan, Republic of Korea. The bacteria were originally sourced from diseased olive flounder and cultured in tryptic soy agar (TSA, Sigma) plates (24 h at 27 °C). All fish were subjected to intraperitoneal injection (i.p.) with 50 μL of *E. tarda* (3 × 10^8^ CFU/mL) solution. The water temperature was maintained at 25 ± 1.0 °C (mean ± SD) and during 13th day of the challenge test, and mortalities were recorded daily from each tank. Dead fish were necropsied and kidney samples were taken and streaked on SS agar (Difco, Birmingham, UK). Black pigments confirmed *E. tarda* infection.

### 2.7. Real-Time PCR

Tissue fragments from hexokinase (HK) were obtained and immediately stored at −80 °C in TRIzol reagent (Thermo Fisher Scientific, San Jose, CA, USA) for RNA extraction. Total RNA was extracted from 0.5 g of olive flounder tissue using TRIzol Reagent (Thermo Fisher Scientific). Afterwards, it was quantified and the purity was assessed spectrophotometrically. The RNA was then treated with DNase I (Cosmogenetech, Seoul, Republic of Korea) to remove genomic DNA contamination. Complementary DNA (cDNA) was synthesized using M-MuLV reverse transcriptase (Cosmogenetech). The expression of four selected immune-relevant genes, including FGH (flounder growth hormone), IL-1β (interleukin 1β), IL-10 (interleukin 10), and β-actin (beta-actin as house-keeping gene), were analyzed via real-time PCR, which was performed using a Bio-Rad CFX96 (Bio-Rad, Hercules, CA, USA) with SYBR Green PCR Core Reagents (Cosmogenetech). The relative expression levels of the target gene transcripts (*FGH*, *IL-1B*, *IL-10*), with β-actin as an internal control, were calculated using CFX manager software version 2.0 (Bio-Rad) (Table 3). In all cases, each PCR was performed with triplicate samples.

### 2.8. Histology

The histological investigation in the present study was based on previously described work by Choi et al. [36]. Briefly, the distal parts of the anterior intestine tissue of the fish (n = 5 fish per tank) were dissected and immediately fixed in 10% neutral buffered formalin. Then, the tissues were dehydrated in a graded ethanol series and embedded in paraffin. Tissue blocks were sectioned (4 μm thickness) using a microtome machine (HistoCore, Leica Biosystems, Buffalo Grove, IL, USA) and stained with hematoxylin and eosin (H&E). Tissue sections were examined under an AX70 Olympus (Tokyo, Japan) microscope adjusted with a digital camera (DIXI Optics, Daejeon, Republic of Korea). The tissue images were analyzed using Image J 1.32j software (National Institute of Health, Bethesda, MD, USA) where at least 6 images per slide were taken into consideration for statistical analyses.

### 2.9. Digestive Enzyme

Intestinal trypsin, amylase and lipase activity was measured using a trypsin activity colorimetric assay kit (BioVision, Exton, PA, USA). According to manufacturer’s instructions, 100 mg sections of intestine were taken, homogenized using trypsin assay buffer, and centrifuged at 5000× *g* for 10 min. Clear samples from the upper sections of the tubes were mixed with trypsin inhibitor, and the optical density was read at 405 nm in 0, 20, 40, 60, 80, 100 and 120 min (Sunrise TECAN, Männedorf, Switzerland). Trypsin activity was defined as the amount of enzyme that catalyzed the conversion of 1 μmol of substrate per minute per mU (mU/mL).

### 2.10. Statistical Analysis

The mean values of fish tank replicates (n = 3) were used for statistical analysis. Normality and homogeneity of variance were assessed for all data using the Shapiro–Wilk and O’Brien tests, respectively. All the data were analyzed via one-way ANOVA (Statistix 3.1; Analytical Software, St. Paul, MN, USA) to test the effects of the dietary treatments. When a significant treatment effect was observed, an LSD test was used to compare the means. Treatment effects were considered significant at *p* < 0.05. A survivability curve for the challenge test was established based on Kaplan and Meier [40] to determine the differences in survival rates among the treatment groups.

## 3. Results

### 3.1. Growth Performance

The growth performances of juvenile olive flounder fed different experimental diets for 8 weeks are presented in Table 4. At the end of feeding trial, the WG and SGR of fish fed the GRO, GROMW, GROPT and GROTW diets were significantly higher than those of fish fed CON, TW and OTC diets (*p* < 0.05). The FE of fish fed the GROMW and GROTW diets was significantly higher than those of fish fed PT, TW, MW and OTC diet (*p* < 0.05). The PER of fish fed the GRO, GROMW and GROTW diets were significantly higher than those of fish fed the CON, TW and OTC diet (*p* < 0.05). However, the survival rate, HSI, VSI and CF of fish were not significantly affected by the experimental diets (*p* > 0.05).

### 3.2. Whole-Body Proximate Composition

There were no significant differences in the crude protein, lipid, moisture and ash content among the group of fish fed different experimental diets for 8 weeks (Table 5).

### 3.3. Hematological Parameters

The blood parameters of juvenile olive flounder fed the experimental diets are shown in Table 6. There were no significant differences in the GOT, glucose and total protein content among the group of fish fed different experimental diets for 8 weeks. However, the GPT of fish fed the GROPT diet was significantly lower than that of fish fed the CON diet (*p* > 0.05).

### 3.4. Non-Specific Immune Responses

The non-specific immune responses of juvenile olive flounder fed different experimental diets for 8 weeks are shown in Table 7. The superoxide dismutase (SOD) activity of fish fed the PT, TW GRO, GROMW, GROPT, GROTW and OTC diets was significantly higher than that of fish fed the CON and MW diets (*p* > 0.05). The lysozyme activity of fish fed the PT, GRO, GROMW, GROPT, GROTW and OTC diets was significantly higher than that of fish fed the CON and TW diets (*p* > 0.05). The myeloperoxidase activity of fish fed the TW, GRO, GROMW, GROPT, GROTW and OTC diets was significantly higher than that of fish fed the CON, PT and MW diets (*p* > 0.05).

### 3.5. Growth- and Immune-Related Gene Expressions

**Figure 1:**
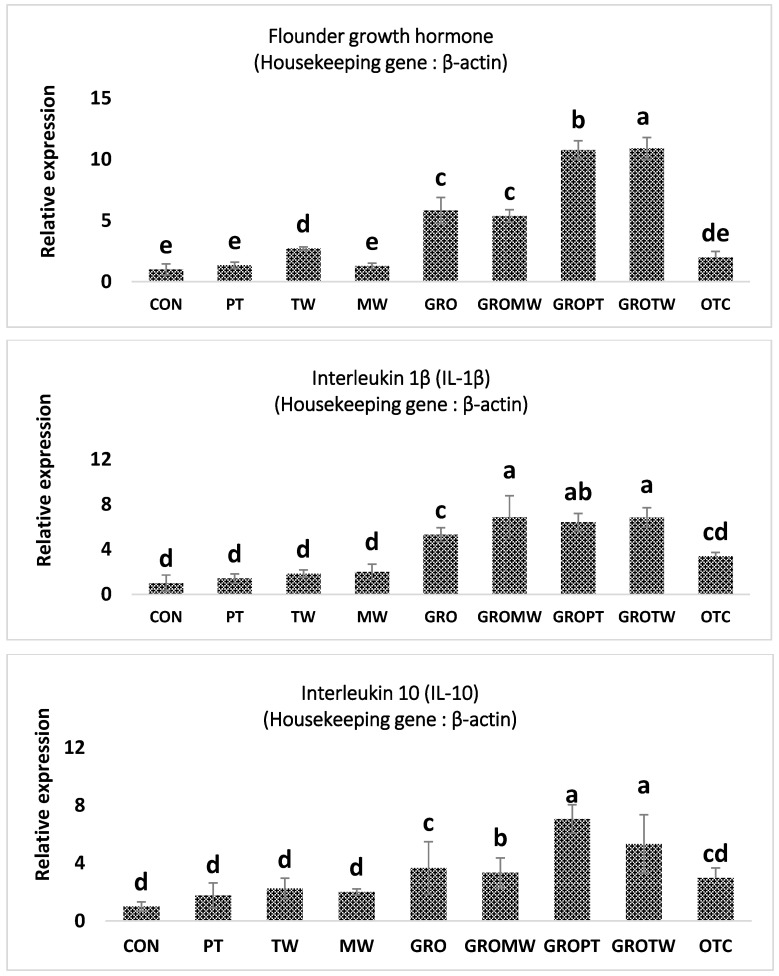
Relative mRNA expression levels of flounder growth hormone (*FGH*), interleukin-1 beta (*IL-1β*) and interleukin-10 (*IL-10*) of intestines of olive flounder fed the nine experimental diets for 8 weeks. Values are means from triplicate groups of fish, where the values on each bar with different superscripts (a, b, c, d, e) are significantly different (*p* < 0.05).

The gene expressions of the immunological parameters in the intestines of olive flounder fed diets supplemented with feed additives are presented in Figure 1. The mRNA activity of flounder the growth hormone gene (FGH) of fish fed the TW, GRO, GROMW, GROPT and GROTW diets was significantly higher than that of fish fed the CON, PT and MW diets (*p* < 0.05). Moreover, interleukin-1β (IL-1β) and interleukin 10 (IL-10) expression of fish fed the GRO, GROMW, GROPT and GROTW diets was significantly higher than that of fish fed the CON, PT, TW and MW diets (*p* < 0.05). Meanwhile, fish fed the GROTW diet showed higher FGH, IL-1β and IL-10 expression compared to fish fed all the other experimental diets (*p* < 0.05).

### 3.6. Histology

**Figure 2:**
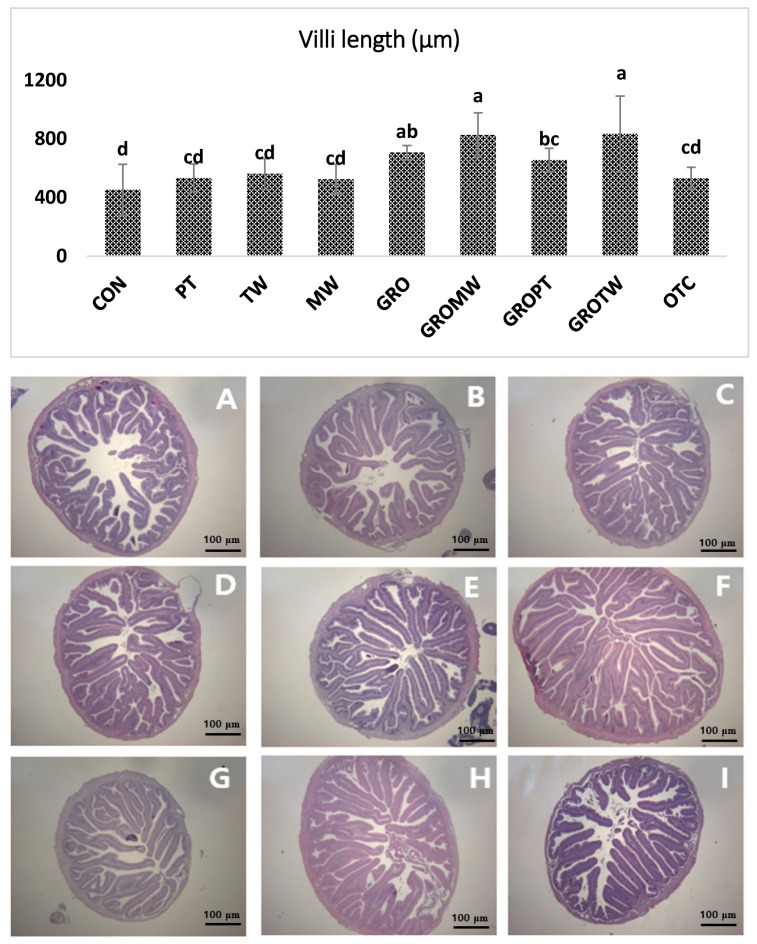
Intestinal histology of juvenile olive flounder fed the nine experimental diets ((**A**) CON, (**B**) PT, (**C**) TW, (**D**) MW, (**E**) GRO, (**F**) GROMW, (**G**) GROPT, (**H**) GROTW and (**I**) OTC) for eight weeks (scale bar = 100 μm; original magnification ×40). Regarding villi length (upper graph), values are means from triplicate groups of fish, where the values on each bar with different superscripts (a, b, c, d) are significantly different (*p* < 0.05).

The results of the histological analysis of the anterior intestines of olive flounder fed different experimental diets for 8 weeks are shown in Figure 2. Fish in the group of GRO, GROMW, GROPT and GROTW clearly exhibit better intestinal histomorphology with more massive villi compared to other treatment groups. In addition, A and I (Figure 2) show certain untidiness and improper arrangement of villi compared to the other groups.

### 3.7. Digestive Enzyme

**Figure 3:**
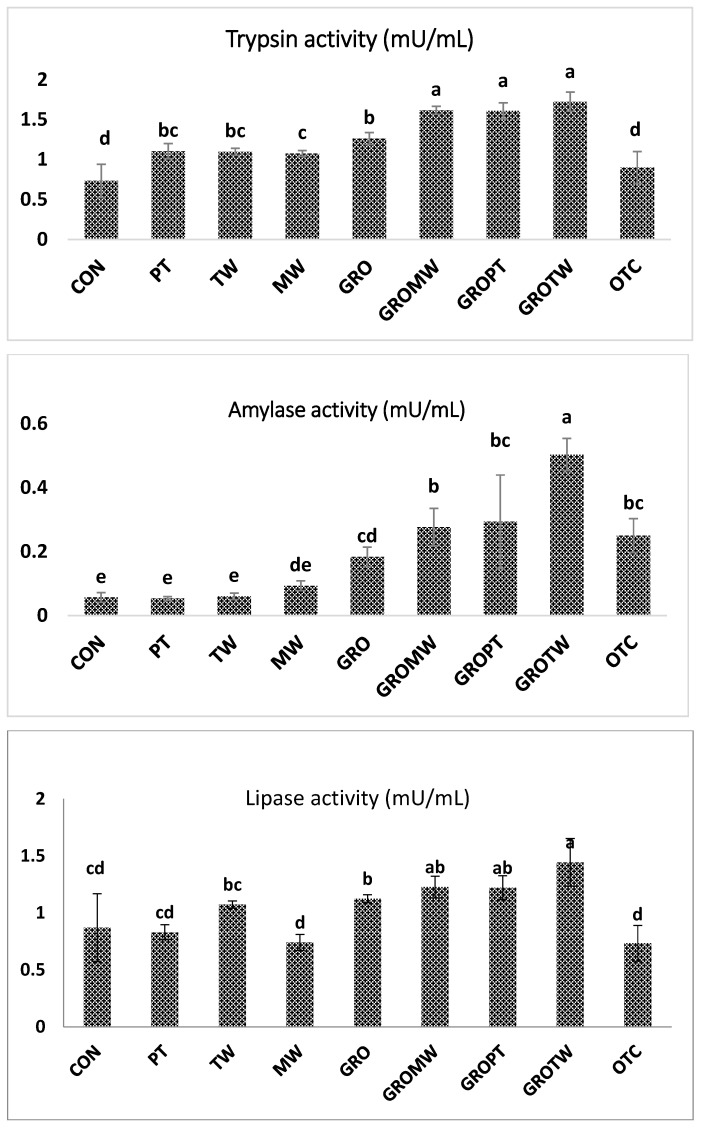
Digestive enzyme activities of juvenile olive flounder fed the nine experimental diets for eight weeks. Values are means from triplicate groups of fish, where the values on each bar with different superscripts (a, b, c, d, e) are significantly different (*p* < 0.05).

The intestinal trypsin, amylase and lipase activity (mU/mL) of juvenile olive flounder fed the nine experimental diets is shown in Figure 3. Trypsin activity was significantly higher for fish fed the PT, TW, MW, GRO, GROMW, GROPT and GROTW diets compared to those of fish fed the CON and OTC diets (*p* < 0.05). Meanwhile, the trypsin activity of fish fed the synergetic diet was significantly higher than in all the other groups (*p* < 0.05). Amylase activity was significantly higher for fish fed the GRO, GROMW, GROPT, GROTW and OTC diets compared to that of fish fed the CON, PT and TW diets (*p* < 0.05). Lipase activity was significantly higher for fish fed the TW, GRO, GROMW, GROPT and GROTW diets compared to that of fish fed the MW and OTC diets (*p* < 0.05).

### 3.8. Challenge Test

**Figure 4:**
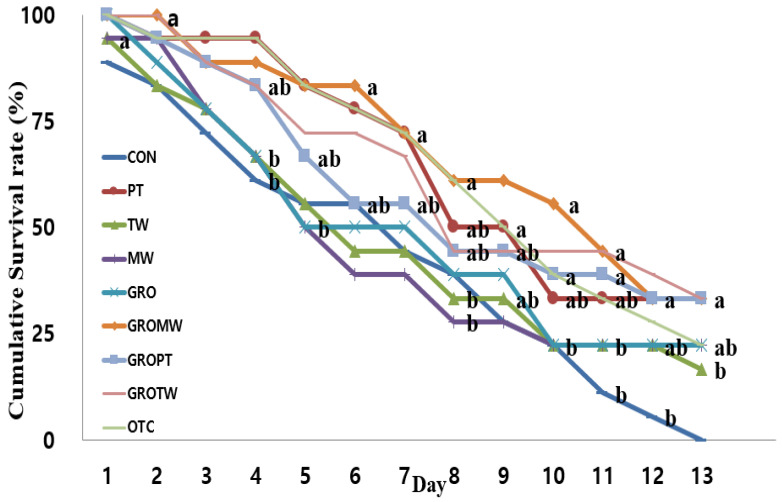
Cumulative survival rate after challenge with *E. tarda* for 13 days in olive flounder fed the nine experimental diets for 8 weeks. Values are means from triplicate groups of fish, where the values on each bar with different superscripts (a, b) are significantly different (*p* < 0.05).

Cumulative survival rate of juvenile olive flounder challenged with *E. tarda* for 13 days is shown in Figure 4. During the challenge test, the first mortalities occurred on the second day, and mortality was pronounced after the third day of injection. At the end of 13 days of the challenge test, the cumulative survival rate of fish fed the PT, GROMW, GROPT and GROTW diets was significantly higher than that of fish fed CON, TW, MW and OTC diets (*p* < 0.05).

## 4. Discussion

The experiment showed that the diet containing a yeast extract nucleotide (GRO) and the diet combining a nucleotide and other feed additives had higher WG, SGR and PER than the control. These results are supported by intestinal histology and growth (FGH) gene expression. The results of this experiment were similar to those of Xiong et al. [41]. Yeast extracts contain low-molecular-weight intracellular compounds, nucleotides, nucleosides, peptides and ß-glucans [42]. Nucleotides and nucleosides have not been considered essential nutrients due to their endogenous supply through de novo metabolic synthesis and the salvage pathways [33]. However, during early life stages, stressful conditions, injuries and nutrient deficiencies, an exogenous supply of nucleotides seems necessary [18]. In this study, the peptide and taurine diets showed significantly higher non-specific immune responses than control diet. The addition of taurine to feed is reported to improve the growth performance of many fish species. Kim et al. [43] reported that the taurine requirement of olive flounder is 1.0% for juveniles at an optimal water temperature. In this experiment, the optimal water temperature was maintained during the experimental period, and we used juvenile olive flounder. Many studies have been reported on whether taurine has a positive effect on fish immunity. Fang et al. [44] reported that taurine can directly scavenge free radicals. Higuchi et al. [45] also showed elevated SOD levels in eels fed a taurine-supplemented diet. In addition, studies by Kim et al. [46] and Han et al. [47] have shown that the addition of taurine to olive flounder feed increases the non-specific immune response. Interestingly, the present study also showed that SOD and MPO levels were significantly increased in fish diets with taurine supplementation. Lysozymes are an important defense enzyme of the innate immune system of fishes [48] and has been used as a key parameter to evaluate non-specific defense ability [49,50]. Peptide is a substance extracted from yeast. Yeast extract is a non-antibiotic functional product that is naturally obtained from yeast strains such as *Saccharomyces cerevisiae* and *Kloeckera apiculata* [51]. Although the composition of yeast extracts is variable, they essentially contain some nucleotides and β-glucans, which are immunostimulatory and possibly antimicrobial [52,53]. According to a study by Gao et al. [54], the addition of peptides to chicken feed in quantities of 2.5 g/kg showed significantly higher levels of lysosomal activity and growth compared to the controls.

Growth hormone is a hormone that stimulates the secretion of IGF-1 in the liver and increases the concentration of glucose and vitreous acid [55]; the produced IGF-1 induces protein synthesis [56], and is reported as a chemical indicator of growth factors in fish, such as those promoting cell division [57]. In the present experiment, olive flounder fed taurine (TW), nucleotide (GRO) and a combination of nucleotide and other feed-additive-supplemented diets exhibited a significantly higher expression of FGH than those fed the control, peptide and mineral water diets. This result is in line with other studies in which the addition of nucleotide showed high values in FGH [58]. Cytokine is a polypeptide or glycoprotein that is secreted from various kinds of cell in the body and is involved in cell proliferation, differentiation, activation, etc., and plays an important role in immune and inflammatory reactions, and there is a protein factor called interleukin in these cytokines. Most of these factors are known to be involved in promoting growth or enhancing cellular immune activity, promoting the selective proliferation of immune cells, activating macrophages, allergies, and anticancer activities [59]. Ryu et al. [60] reported that an interleukin is associated with immunostimulants at the animal testing level through a mixture of various extracts, especially when seaweeds such as *Hizikia fusiformis* are eaten [60,61,62]. In this experiment, IL-1β, IL-10 yeast extract nucleotide (GRO) and the diet combining nucleotide and other feed additives had significantly higher results than the control, peptide, taurine and mineral water diets. Based on the above results, the increase in interleukin, an immune-promoting factor, is believed to have been identified when various additives were used.

The intestinal parameters, especially villi length, are integral parts of the gastrointestinal tract (GIT) and represent the gut health status of fish. The GIT helps to absorb and transfer the nutrients from gut to the organs through blood circulation after digestion and absorption of the nutrients in the diet. Therefore, a larger surface area and height of the intestinal villus means more absorption of nutrients in the gut. Histological sections of the distal intestine of olive flounder are shown in Figure 2. In the present experiment, diets supplemented with nucleotide (GRO) and the combination of nucleotide with other feed additives exhibited a significantly higher surface area and more elongated villi than the control diet, which might be attributed to the better health status of the GIT as well as higher absorption of nutrients in the GIT. Our previous experiments showed that the length of the intestinal villus increased in juvenile olive flounder fed with nucleotides [36]. In agreement with the present study, Siddik et al. [63] reported higher villi length following the oral administration of a yeast-supplemented diet than a control diet in barramundi fish. Therefore, in the present study, dietary yeast-extracted nucleotides, alone or in combination with other additives, confirmed that there are more functional and active intestinal absorptive cells in the intestines.

Enzymes are an important group of feed additives due to their specific role in the digestion of plant-origin feedstuff [18]. It was shown that the digestive enzyme activity of fish fed a diet supplemented with nucleotides and the combination of nucleotides with other feed additives exhibited significantly higher results than those fed the control diet. It has been previously reported that addition of feed additives to fish diets can improve digestibility and gut health and lead to increased growth performance, especially in low-fish-meal diets [21].

Additionally, the disease resistance properties of dietary nucleotides against pathogenic bacteria, viruses and parasites have been previously demonstrated [42], although these mechanisms are not clearly understood. In the present study, the cumulative survival curve showed a positive impact of dietary yeast-extracted nucleotides with or without the combination of mineral water, peptide or taurine supplements in juvenile olive flounder after 13 days of the challenge test. These results align with the growth, serological and immune parameters of this study.

## 5. Conclusions

In conclusion, the results of the present study demonstrate that dietary yeast-extracted nucleotides, alone or in combination with mineral water, peptide or taurine, could replace antibiotics without affecting growth and immune status in juvenile olive flounder. The results of the present study could be helpful in overcoming the overwhelming dependence on fish meal in the aquaculture industry. Furthermore, the results of the present study could be beneficial in terms of the cost-effective formulation of aquafeeds with low amounts of fish meal for the sustainable development of olive flounder aquaculture.

## Figures and Tables

**Table 1:** Composition of the basal diet (% of dry matter basis).

Ingredients	Percentage (%)
Fish meal, sardine ^1^	22.5
Fish meal, anchovy ^1^	22.5
Soybean meal	12.0
Starch ^2^	3.8
Wheat gluten meal ^2^	4.5
Soy protein concentrate	5.5
Tankage meal	8.0
Poultry by-product meal	4.5
Wheat flour	7.0
Fish oil ^3^	4.3
Vitamin premix ^4^	1.2
Mineral premix ^5^	1.2
Others ^6^	3.0
*Proximate analysis* (% of dry matter basis)	
Moisture	8.2
Crude protein	54.8
Crude lipid	9.4
Crude ash	10.4

^1^ Suhyup Feed Co., Uiryeong, Republic of Korea. ^2^ The feed Co., Goyang, Republic of Korea. ^3^ Jeil feed Co., Hamman, Republic of Korea. ^4^ Contains (as mg/kg in diets) ascorbic acid, 300; DL-calcium pantothenate, 150; choline bitate, 3000; inositol, 150; menadione, 6; niacin, 150; pyridoxine hydrochloride, 15; riboflavin, 30; thiamine mononitrate, 15; DL-α-Tocopherol acetate, 201; retinyl acetate, 6; biotin, 1.5; folic acid, 5.4; cobalamin, 0.06. ^5^ Contains (as mg/kg in diets) NaCl, 437.4; MgSO_4_·7H_2_O, 1379.8; ZnSO_4_·7H_2_O, 226.4; Fe-citrate, 299; MnSO_4_, 0.016; FeSO_4_, 0.0378; CuSO_4_, 0.00033; Ca(IO)_3_, 0.0006; MgO, 0.00135; NaSeO_3_, 0.00025. ^6^ calcium phosphate, lecithin, betaine, taurine, choline, vitamin C, vitamin E.

**Table 2:** Proximate composition of the nine experimental diets (% of dry matter basis) ^1^.

Item	Diets (%)								
	CON	PT	TW	MW	GRO	GROMW	GROPT	GROTW	OTC
Moisture	8.19	8.08	8.20	8.21	8.23	8.21	8.15	8.16	8.23
Crude protein	54.8	54.5	54.2	54.1	54.8	54.7	54.6	54.5	54.2
Crude lipid	9.39	9.12	9.02	9.12	9.06	9.29	9.31	9.23	9.21
Crude ash	10.4	10.2	10.4	10.4	10.6	10.9	10.1	10.4	10.1

^1^ PT (peptide, 3 g/kg), TW (taurine, 5 g/kg), MW (mineral water, 217 ppm/kg), GRO (Gro-pro, 4 g/kg), GROMW (Gro-pro, 4 g/kg + mineral water, 217ppm/kg), GROPT (Gro-pro, 4 g/kg + peptide, 3 g/kg), GROTW (Gro-pro, 4 g/kg + taurine, 5 g/kg), OTC (oxytetracycline, 5 g/kg).

**Table 3:** Primer sequences, amplicon lengths and gene bank accession numbers used for qPCR assays (5′→3′) in this study.

Name of Gene	Sense	Oligonucleotide Sequence (5′→3′)	Amplicon Length (bp)	Gene Bank Accession Number
*β-actin*	Forward	CAGCATCATGAAGTGTGACGTG	107	HQ386788.1
	Reverse	CTTCTGCATACGGTCAGCAATG		
*FGH* ^1^	Forward	CGCCGTATGGAAACTCTGAACT	160	M23439.1
	Reverse	GGGTGCAGTTAGCTTCTGGAAA		
*IL-1β* ^2^	Forward	ATGGAATCCAAGATGGAATGC	250	KF025662.1
	Reverse	GAGACGAGCTTCTCTCACAC		
*IL-10* ^3^	Forward	AGCGAACGATGACCTAGACACG	114	KF025662.1
	Reverse	ACCGTGCTCAGGTAGAAGTCCA		

^1^ FGH: flounder growth hormone; ^2^ IL-1β: interleukin 1 beta; ^3^ IL-10: interleukin 10.

**Table 4:** Growth performance of olive flounder fed the nine experimental diets for 8 weeks ^1^.

Item	Diets									Pooled SEM ^12^
	CON	PT	TW	MW	GRO	GROMW	GROPT	GROTW	OTC	
IBW ^2^	5.20 ^ns^	5.19	5.19	5.16	5.12	5.15	5.09	5.16	5.11	0.01
FBW ^3^	22.9 ^d^	23.6 ^bcd^	23.1 ^cd^	24.9 ^abcd^	25.5 ^ab^	25.8 ^a^	25.1 ^abc^	26.9 ^a^	23.1 ^cd^	0.48
WG (%) ^4^	340 ^d^	354 ^bcd^	345 ^cd^	382 ^abcd^	398 ^ab^	402 ^a^	392 ^abc^	422 ^a^	352 ^cd^	9.76
SGR (%/day) ^5^	2.69 ^c^	2.75 ^bc^	2.71 ^c^	2.86 ^abc^	2.91 ^ab^	2.93 ^a^	2.90 ^ab^	3.00 ^a^	2.74 ^c^	0.04
FE (%) ^6^	122 ^bcd^	114 ^cd^	109 ^d^	118 ^cd^	124 ^bc^	132 ^ab^	127 ^abc^	139 ^a^	114 ^d^	3.21
PER ^7^	1.75 ^c^	1.82 ^bc^	1.76 ^c^	1.96 ^abc^	2.07 ^ab^	2.07 ^ab^	1.93 ^abc^	2.14 ^a^	1.84 ^c^	0.05
Survival (%) ^8^	97.3 ^ns^	89.3	86.7	86.7	88.0	93.3	96.0	96.0	91.0	1.30
HSI (%) ^9^	0.89 ^ns^	1.24	1.27	0.99	1.27	1.11	1.03	1.26	1.00	0.05
VSI (%) ^10^	1.65 ^ns^	1.85	1.76	1.64	1.72	1.75	1.50	1.76	1.70	0.03
CF ^11^	0.78 ^ns^	0.80	0.79	0.77	0.82	0.76	0.75	0.78	0.76	0.01

^1^ Values are means from triplicate groups of fish, where the values in each row with different superscripts (a, b, c, d) are significantly different (*p* < 0.05), and ns = non-significant; ^2^ initial body weight; ^3^ final body weight; ^4^ weight gain (WG, %) = [(final weight − initial weight) × 100]/initial weight; ^5^ feed efficiency ratios (FE, %) = (wet weight gain/dry feed intake) × 100; ^6^ specific growth rates (SGR, %) = [(log_e_ final weight − log_e_ initial weight) × 100]/days; ^7^ protein efficiency ratio (PER) = (wet weight gain/protein intake); ^8^ hepatosomatic index (HSI) = (liver weight × 100)/body weight; ^9^ viscerosomatic index (VSI, %) = (viscera weight × 100)/body weight; ^10^ condition factor = (wet weight/total length^3^) × 100; ^11^ survival rates = [(total fish − dead fish) × 100]/total fish; ^12^ pooled standard error of means.

**Table 5:** Whole-body proximate composition of olive flounder fed the nine experimental diets for 8 weeks ^1^.

Item	Diets									Pooled SEM
	CON	PT	TW	MW	GRO	GROMW	GROPT	GROTW	OTC	
Moisture	76.3 ^ns^	75.4	74.3	75.9	75.3	75.9	76.3	76.1	75.3	0.24
Protein	17.9 ^ns^	18.4	18.7	17.7	18.6	18.1	18.2	17.8	18.5	0.14
Lipid	1.87 ^ns^	2.08	2.54	2.08	2.32	2.17	1.72	2.23	2.1	0.09
Ash	4.37 ^ns^	4.51	4.55	4.31	4.24	4.34	4.46	4.40	4.55	0.04

^1^ Values are means from triplicate groups of fish, where the values in each row with no superscripts are non-significantly (ns) different (*p* < 0.05).

**Table 6:** Serum biochemistry of olive flounder fed the nine experimental diets for 8 weeks ^1^.

Item	Diets									Pooled SEM
	CON	PT	TW	MW	GRO	GROMW	GROPT	GROTW	OTC	
GOT ^2^	35 ^ns^	27	32	30	31	40	25	25	25	1.69
GPT ^3^	12.7 ^a^	11.0 ^ab^	10.7 ^ab^	10.7 ^ab^	10.3 ^ab^	11.3 ^ab^	8.3 ^b^	9.7 ^ab^	9.7 ^ab^	0.4
GLU ^4^	13.3 ^ns^	11.3	9.7	14.0	13.0	13.3	11.0	11.3	10.3	0.51
TP ^5^	2.6 ^ns^	2.8	2.7	3.1	2.8	3.2	2.3	2.6	2.4	0.1

^1^ Values are means from triplicate groups of fish, where the values in each row with different superscripts (a, b) are significantly different (*p* < 0.05), ns = non-significant. ^2^ Glutamic oxaloacetic transaminase (U/L). ^3^ Glutamic pyruvic transaminase (U/L). ^4^ Glucose (mg/dL). ^5^ Total protein (g/dL).

**Table 7:** Antioxidant enzymes and li of olive flounder fed the nine experimental diets for 8 weeks ^1^.

Item	Diets									Pooled SEM
	CON	PT	TW	MW	GRO	GROMW	GROPT	GROTW	OTC	
SOD ^2^	75.4 ^d^	82.4 ^c^	81.7 ^c^	76.5 ^d^	90.5 ^b^	93.7 ^ab^	97.5 ^a^	95.9 ^ab^	92.4 ^b^	2.81
Lysozyme ^3^	0.77 ^cd^	0.83 ^a^	0.74 ^d^	0.78 ^bc^	0.83 ^a^	0.86 ^a^	0.88 ^a^	0.85 ^a^	0.79 ^b^	0.02
MPO ^4^	1.98 ^d^	2.09 ^d^	2.25 ^c^	2.15 ^d^	2.46 ^c^	2.65 ^ab^	2.87 ^a^	2.25 ^b^	2.19 ^bc^	0.10

^1^ Values are means from triplicate groups of fish, where the values in each row with different superscripts (a, b, c, d) are significantly different (*p* < 0.05); ^2^ SOD (% inhibition): superoxide dismutase activity; ^3^ Lysozyme (U/mL): lysozyme activity; ^4^ MPO (absorbance): myeloperoxidase (OD at 450 nm).

## Data Availability

The raw data supporting the conclusions of this article can be made available by the corresponding author without undue reservation.
